# Label-free single-cell separation and imaging of cancer cells using an integrated microfluidic system

**DOI:** 10.1038/srep46507

**Published:** 2017-04-20

**Authors:** Maria Antfolk, Soo Hyeon Kim, Saori Koizumi, Teruo Fujii, Thomas Laurell

**Affiliations:** 1 Dept. of Biomedical Engineering, Lund University, Lund, Sweden; 2 Biotech Research and Innovation Centre, University of Copenhagen, Copenhagen, Denmark; 3 Dept. of Bioengineering, University of California, Los Angeles, USA; 4 Institute of Industrial Science, The University of Tokyo, Tokyo, Japan; 5 CREST, Japan Science and Technology Agency, Tokyo, Japan; 6 Dongguk University, Seol, South Korea

## Abstract

The incidence of cancer is increasing worldwide and metastatic disease, through the spread of circulating tumor cells (CTCs), is responsible for the majority of the cancer deaths. Accurate monitoring of CTC levels in blood provides clinical information supporting therapeutic decision making, and improved methods for CTC enumeration are asked for. Microfluidics has been extensively used for this purpose but most methods require several post-separation processing steps including concentration of the sample before analysis. This induces a high risk of sample loss of the collected rare cells. Here, an integrated system is presented that efficiently eliminates this risk by integrating label-free separation with single cell arraying of the target cell population, enabling direct on-chip tumor cell identification and enumeration. Prostate cancer cells (DU145) spiked into a sample with whole blood concentration of the peripheral blood mononuclear cell (PBMC) fraction were efficiently separated and trapped at a recovery of 76.2 ± 5.9% of the cancer cells and a minute contamination of 0.12 ± 0.04% PBMCs while simultaneously enabling a 20x volumetric concentration. This constitutes a first step towards a fully integrated system for rapid label-free separation and on-chip phenotypic characterization of circulating tumor cells from peripheral venous blood in clinical practice.

Due to changes in lifestyle factors and an aging population, cancer is becoming more common worldwide^1^. New technological developments have enabled earlier diagnosis opportunities which may improve patient outcome, but the dissemination of the cancer to remote tissues where metastases are formed is still the major cause of cancer deaths^2^. Metastases are developed when cancer cells are shed from the primary tumor into the blood stream, where they travel to other tissues^3^. Cancer cells found in the blood circulation are referred to as circulating tumor cells (CTCs). CTCs have been detected in patient samples from all major cancers that have reached a metastatic stage but only very rarely in healthy subjects^4^,^5^,^6^. These cells has been found in quantities between 1–10 000 CTCs/mL, a number that may vary dependent on the primary tissue that the circulating tumor cell originates from^7^^7^. Finding and monitoring these cancer cells is critical to improve survival of the patient as the number of these cells is prognostic for survival and disease progression in many types of cancers^8^,^9^. Furthermore, isolating CTCs will provide a cell source for targeted drug development and in depth biomolecular analysis of these cells may provide insights to the evolution of the cancer tumor and expected treatment response, paving the way towards a more personalized treatment^4^,^9^.

Much focus has been directed towards developing techniques for enumeration and detection of CTCs, including the CellSearch^®^ system. Most efforts have been focused towards carcinomas and the use of immunolabels specific to epithelial cells, such as EpCAM or different cytokeratins^5^. There is, however, a fear that these epithelial cell markers are lost during the epithelial-mesenchymal transition that the carcinoma cancer cells are thought to undergo to become more motile and escape the primary tumor into the blood stream^10^,^11^,^12^. Label-free methods or methods based on additional markers may therefore be able to detect additional numbers of cancer cells or subpopulations that currently goes undetected.

Microfluidics has been extensively explored for cell separation purposes and much effort has been directed towards CTC isolation where also untraditional biomarkers such as size, shape, compressibility, deformability, or dielectric properties have been explored along with the more traditional immunolables^13^,^14^. All major microfluidic methods have been explored for this purpose, including microfilters^15^ inertia^16^,^17^, deterministic lateral displacement^18^, dielectrophoresis^19^, acoustophoresis^20^,^21^,^22^, affinity chromatography^23^,^24^, magnetophoresis^25^, or combinations thereof 26, which are especially promising as they utilize the combined advantages of more than one method.

A practical challenge for all systems not relying on on-chip capture arises when the sorted target cells should be collected for downstream analysis. In order to recover a sufficient number of cancer cells for subsequent analysis a relatively large volume of blood have to be processed. This commonly results in the collection of the target cells in an equivalent or even larger volume than the input sample volume that has to be prepared for analysis. This in turn imposes a subsequent sample concentration step before analysis, traditionally performed by centrifugation, where sample loss of the already rare cells is a considerable risk. A separation system that could enable direct on-chip analysis would effectively eliminate this post-separation risk of losing target cells. A few systems have been developed that include this option and their analytical performance is summarized in Table 1. (For a more thorough review on the subject of microfluidic rare cell separation see^27^).

These systems all effectively eliminates the risk of post-separation cell losses. However, they show pour recoveries or are label-dependent which induces the risk of loosing subpopulations low in expression of the label. A label-free separation system enabling on-chip analysis would therefore also allow for the collection of a more homogenous sample better representing the *in vivo* CTC population.

Recently, we developed a microfluidic device for rare cell analysis, combining an acoustic chip for sample preconcentration^34^ with a dielectrophoresis (DEP) chip for subsequent single-cell trapping^35^,^36^. Using this device we could show a 10x increase in sample throughput compared to using the DEP-trap alone with a recovery of over 90%^37^. However, this device did not allow for direct processing of clinically relevant samples since a separation function was not integrated. To address this shortcoming, this paper presents a novel integrated device, enabling simultaneous acoustofluidic label-free separation and concentration^38^ of target cells, direct dielectrophoretic single-cell trapping, followed by observation of live cells in a single cell array format. The integrated system demonstrates the separation, concentration and trapping of single live cancer cells without sample transfer between each process, which could be utilized for the development of an automated single-cell CTC analysis system.

## Results and Discussion

In this paper an integrated device capable of simultaneous separation and concentration of cancer cells, followed by single-cell trapping and imaging is realized. The main objective was to minimize rare cell loss in the post-separation sample processing steps, by integrating the separation with the trapping and on-chip analysis.

**Figure 1:**
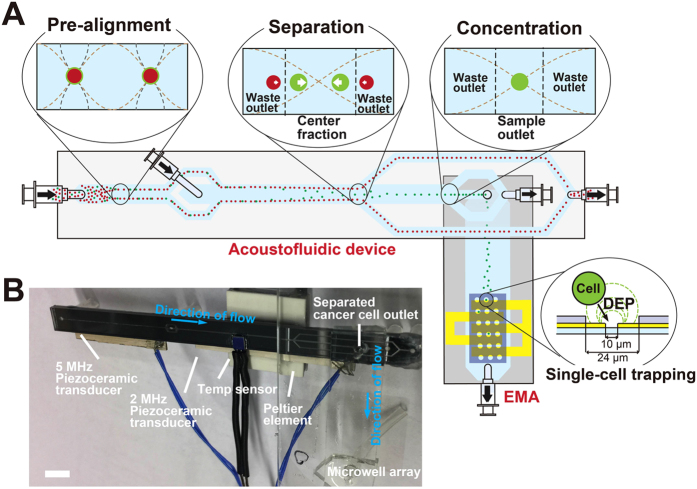
(**A**) The integrated device illustrating the difference operation zones of the acoustophoresis chip and the dimensions of the dielectrophoresis trapping array. White arrows in the separation cross-section indicates the lateral acoustophoretic force F_z_ (S1 Eq.1.) (**B**) Integrated device showing the acoustic chip directly bonded to the DEP chip. Scale bar is 5 mm.

The present system benefited from the integration of two original microfluidic devices having their original functions; A) continuous flow-based acoustophoresis for cancer cell separation and B) dielectrophoresis for single-cell arraying utilizing an electroactive microwell array (EMA) (Fig. 1). The integration of the functions was achieved by directly adapting and connecting the outlet of the acoustic chip to the inlet of the EMA chip. The target cells introduced into the device were pre-aligned and separated from the unwanted cell types based on their acoustic properties^38^. The isolated target cells were refocused in the central fluid fraction while the cell free fraction was discarded into the side outlets, reducing the volume flow rate of the target cell fraction which continued into the EMA chip. The acoustophoresis concentration step was highly important to enable integration of the two original microfluidic devices by adapting the flow rate to the chip-to-chip interface requirements, *i.e.* gearing down the flow rate to a flow rate required for efficient single-cell trapping of the EMA chip. The isolated and concentrated target cells were, then, trapped into the DEP microwells in a single cell array format.

### Design and characteristics of each microfluidic chip

#### Acoustic separation and concentration

**Figure 2:**
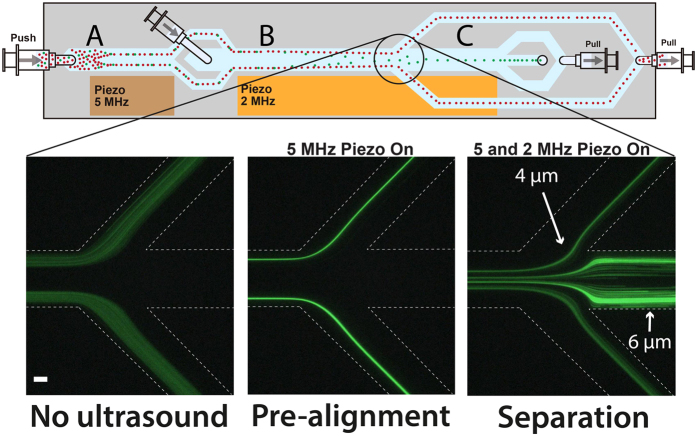
Schematic image of the acoustic chip and images of the separation trifurcation showing the laminated sample with no ultrasound on (left), pre-aligned particles (center) with the 5-MHz piezo on, and separation of the pre-aligned 4 μm and 6 μm fluorescent melamine particles (right) with both the 5- and 2-MHz piezoactuators active. Scale bar indicates 100 μm.

The acoustic chip was used to pre-align, separate, and concentrate the target cells before entering the EMA chip zone (Fig. 1). The pre-alignment channel was used to two-dimensionally preposition all cells into two positions in the acoustic pressure minima located at a distance of ¼ channel width away from each side-wall, and in the middle along the channel height (Fig. 2A). The sample inlet flow rate was set to 80 μL/min which corresponded to the maximum flow rate where all cells were still completely focused in the pre-alignment zone.

The pre-alignment was used to position the cells into the same fluid velocity regime, thereby giving all cells the same lateral starting position as well as retention time in the acoustic separation zone. This ensured an optimal separation performance as opposed to separating cells that are randomly distributed throughout the channel cross-section, which only results in a modest separation efficiency^22^.

The pre-alignment further ensured that the cells’ lateral position at any time in the acoustic field was only defined by its acoustic mobility, which is dependent on the cell size, fluid viscosity, as well as the cell density, and compressibility relative the suspending liquid as defined by the acoustic radiation force, F_z_, acting on the cells (Supplementary Material S1).

The pre-aligned sample entered the acoustophoretic separation zone laminated to the channel walls by a cell-free sheath fluid that was infused in the channel center (Fig. 2B) to increase the separation efficiency. With a starting position closer to the channel wall the cells will have a longer distance to cover to reach the channel center, and will thus also allow two different cells to separate further^21^.

While the smaller cells were diverted to the waste outlet, the larger target cells continued along the channel to the concentration part (Fig. 2C). Here, the cells were re-focused into a single acoustic pressure node in the channel center and collected in the outlet leading them to the trapping zone, while cell-free excess fluid was taken out from the sides. As the separation and concentration was driven by the same piezoceramic transducer the flow rates in the separation and concentration zones had to be optimized together to allow for both optimal separation as well as refocusing in the concentration zone at the same applied acoustic energy. The optimal flow rate for the separation zone was adjusted to 160 μL/min while the flow rate in the concentration zone was set to 10 μL/min in total. The flow rate of the concentration zone was considerably lower than what had previously been used^38^ despite now using a longer channel. This seemingly reduced performance was attributed to the bonding of the rigid acoustic chip, fabricated in silicon and glass, with the softer dielectrophoresis chip fabricated in PDMS, which leads to an increased dissipation of acoustic energy from the silicon/glass chip into the PDMS, making the concentration zone, situated directly along the bonded part of the device, less efficient. Even so, as long as the flow rate was properly adjusted the concentration efficiency was not seen to be affected by this, despite the lower flow rate in the concentration zone.

The optimal frequencies for pre-alignment, separation and concentration were determined using melamine microparticles of 4 μm and 6 μm in diameter suspended in the DEP buffer. Figure 2 shows images of the particle separation trifurcation where the smaller particles are lead off to the sides and the larger particles continue to the concentration zone. The left image shows the particle sample laminated to the channel sides by the particle-free sheath fluid infused in the channel center with no ultrasound turned on. The center image shows the pre-aligned particles without the acoustic separation activated. Finally, the right image shows the separation of the pre-aligned 4 μm and 6 μm melamine particles where the larger, 6 μm particles continue to the concentration zone to be subsequently re-focused while the flow splitter routes off smaller 4 μm particles to the sides, subsequently collected in the waste outlet. As the acoustic migration velocity is proportional to the square of the particles diameter the larger 6 μm particles moves with 2.25 times the velocity of the smaller 4 μm particles and can be effectively separated.

#### Dielectrophoresis trapping

The electroactive microwell is a cell-sized well containing transparent indium tin oxide (ITO) electrodes at the bottom to actively trap single cells using dielectrophoresis (DEP). Since the electric field was highly localized inside of the microwells, a single cell was efficiently attracted into a microwell and a second cell was prevented from entering the microwell due to space restrictions and exposure to Stokes’ drag. The present EMA chip had a large number of microwells, with 5,700 wells covering a 15 mm by 3.8 mm area for high cell-trapping capacity. This constitutes three times more microwells compared to a previous version^37^. The number of microwells in the stream wise direction was increased to efficiently capture the cells that entered from the acoustic chip. A merged image of the whole array is shown in the Supplementary Material S2.

**Figure 3:**
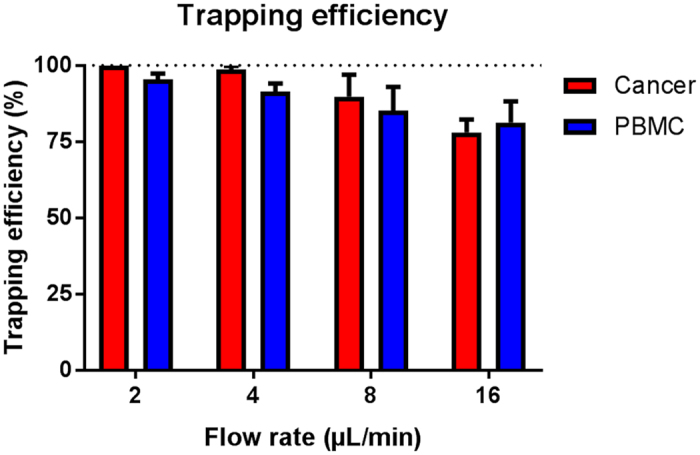
Cell trapping efficiency at different flow rates. The error bars show the standard deviation.

To trap single cells in the microwell array, the DEP force acting on a cell should be balanced to or larger than the Stokes’ drag force induced by the bulk flow used for cell delivery. The trapping efficiency’s (a percentage ratio of the number of trapped cells to the number of cells passing over the microwell array) dependence on the flow rate was evaluated. Dilute DU145 cells, and peripheral blood mononuclear cells (PBMCs) were introduced into the EMA chip and trapped into the microwells by applying an electrical potential of 5 V at 5 MHz with varying flow rates of 2, 4, 8, and 16 μL/min. After a specific time, the number of trapped cells were counted and compared to the cells that were not trapped to calculate the cell trapping efficiency (Fig. 3). A higher flow rate, gives a lower cell trapping efficiency since the stronger drag force does not allow the cells to spend sufficient time in the trapping region for the DEP force to overcome the drag force. Cells initially trapped were also seen to be pulled away in some cases due to a strong drag force.

Hence, an appropriate flow rate was highly required for the efficient single cell trapping. This should not exceed 4 μL/min to ensure optimal trapping conditions. It can be noted that the cells that did not trap were the smaller cells, which is in agreement with the fact that the dielectrophoretic migration velocity is proportional to the square of the diameter of cell^39^. In the tumor cell separation experiments these smaller cells will not enter into the trapping zone as they will be separated into the waste outlet in the acoustic chip. This implies that the trapping efficiency of the separated cells could be expected to be even higher.

### Integrated device performance characterization with dilute live cell sample

The two acousto- and dielectrophoresis chips were integrated by permanently bonding the glass side of the acoustophoresis chip directly to the PDMS of the dielectrophoresis chip, aligning the target cell outlet of the acoustophoresis chip with the inlet of the EMA chip. The cell concentration of whole blood is too high to be processed in a force field based separation method without compromising the separation efficiency since hydrodynamic interactions between the cells would result in target cells pulling unwanted cells into the target cell outlet. Therefore, the combined acoustic separation and DEP trapping system was evaluated on a mixture of tumor cells and peripheral blood mononuclear cells (PBMCs). To be able to characterize the integrated device without immediately filling up the whole trapping array with cells, a highly dilute sample was used with a mixture of viable cells comprised of the prostate cancer cell line DU145 and PBMCs at a concentration of approximately 1000 cells/mL. The PBMCs were obtained through density gradient centrifugation of a blood sample to efficiently deplete the red blood cells. The isolated PBMC fraction was then subsequently spiked with the cancer cells.

**Figure 4:**
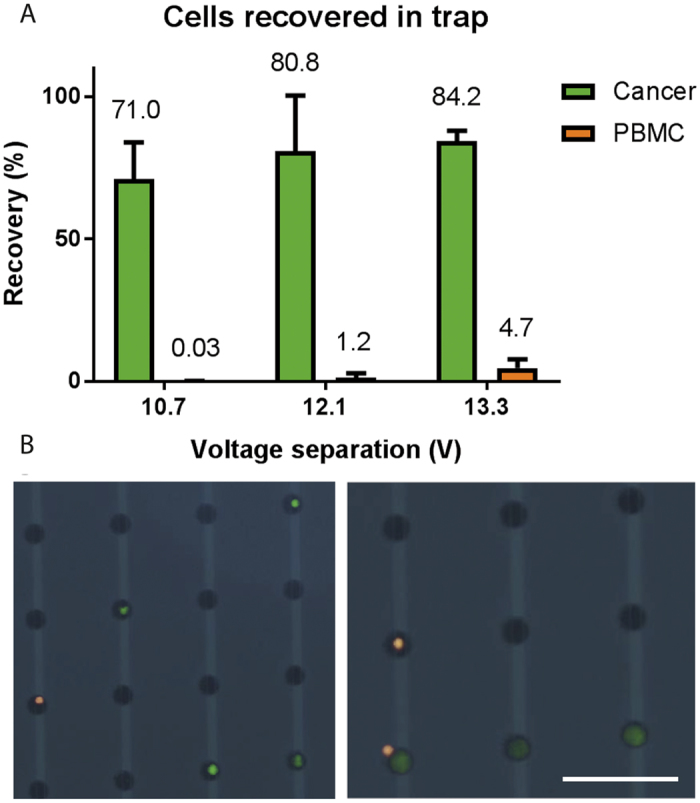
Separation and trapping of dilute cell suspensions of cancer cells and PBMCs. (**A**) The percentage of cells trapped after separation as compared to the input sample. (**B**) Images of trapped cancer cells (green) and white blood cells (orange) that can be directly identified and distinguished in the trap. Scale bar indicated 100 μm. The error bars show the standard deviation.

This sample, with a known concentration of both cell types, was run through the device with the ultrasound turned on for a specific time, corresponding to a defined volume throughput, after which the number of trapped cells were counted to give the recovery. Pre-staining of the cells enabled direct identification and enumeration of the different cells types in the trap using a fluorescent microscope and without the need for more advanced analysis methods. The ordered trapping of the cells in the predefined wells enabled rapid image based identification and recovery estimation of the tumor cells and PBMCs respectively. The results are shown in Fig. 4. During these experiments the voltage of the acoustic pre-alignment transducer was fixed and the voltage of the acoustic separation transducer was varied to recover more or less of the cells into the DEP trapping array. Even though the acoustophoresis concentration zone was actuated using the same transducer, the varied separation voltage was sufficient for all settings to re-focus and concentrate the cells in this zone without losses in the concentration zone. As expected the results show that the transition voltage for the cancer cells to enter the trapping zone is lower than for the PBMCs. At 10.7 volts applied 71.0 ± 12.8% of the cancer cells could be recovered in the trap with only a minute contamination of 0.03 ± 0.1% of the PBMCs. Out of ten runs nine resulted in no contamination of PBMCs at all, which shows that at a cancer cell recovery in the range of 70% a purity of 100% can be achieved using the combined acoustophoretic separation and DEP-trapping system. This is something that is not possible to achieve in most other systems. By applying an even higher voltage on the separation transducer more cancer cells can be recovered at the expense of a decreased purity. Although the cancer cells are larger than the PBMCs and the population size distributions are not overlapping (previously measured to be 15–25 μm in diameter for the cancer cells and 7–14 μm for the white blood cells^21^) they show partly overlapping acoustic mobility and could thus not be perfectly separated. Even so, given the size differences of the two cell populations the separation is predominantly based on size, even if differences in cell density or compressibility has an influence on the acoustophoretic mobility as seen in the fact that the populations cannot be completely separated.

**Figure 5:**
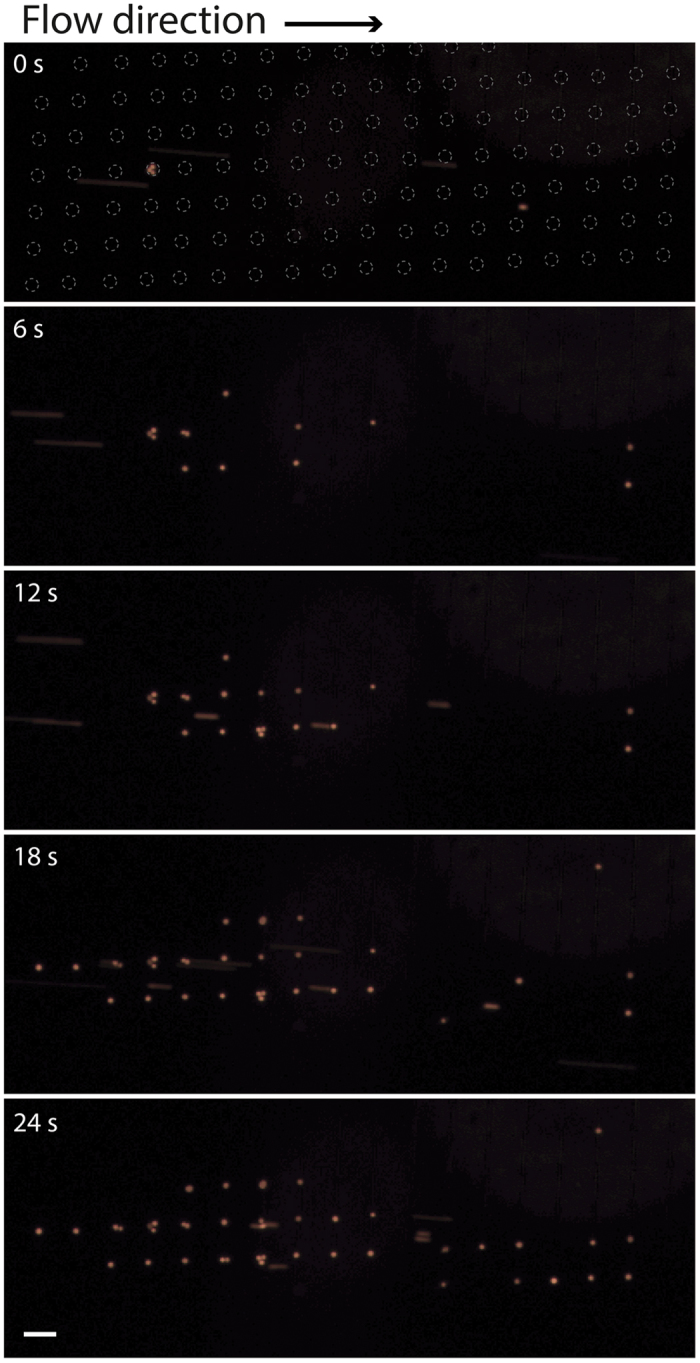
Time lapse sequence showing the trapping of cancer cells (orange) after sorting (extracted from supplementary video S3). The dashed rings at time 0 s indicated the location of the individual single-cell wells. Scale bar indicates 100 μm.

During all the experiments the sample input flow rate was set to 80 μL/min and the flow rate of target cells to the dielectrophoresis trap was 4 μL/min to ensure optimal trapping conditions. In this way, the acoustophoresis concentration step, which geared down the flow rate at no cell loss, was vital for the performance of the integrated device. The lowered flow rate of the target cells entering the trapping zone compared to the sample input flow rate corresponded to a volume concentration of 20x. During the experiments the trapping efficiency of the cancer cells was observed to be 100% even though the initial trapping efficiency experiment indicated that a minute cell loss could occur at this flow rate. The slightly higher trapping efficiency of the pre-separated cancer cells was attributed to the fact that these cells constitute the largest cells of the cancer cell population and thus, as the DEP force is highly dependent on the cell size the trapping efficiency of these cells should be higher compared to the trapping efficiency of the whole spiked cancer cell population. The trapping efficiency will also be dependent on the number of cells previously trapped and will decrease when the trap is approaching saturation. Trapping of separated cancer cells are shown in a time lapse sequence (Fig. 5) extracted from Supplementary Video S3 and images of individually trapped cells are shown in Fig. 4B. Occasionally more than one cell was seen to co-localize in the same well, seen in Fig. 4B (right) where a smaller white blood cell is trapped together with a cancer cell. The occasional trapping of more than one cell in the same well is dependent on the level of the dielectrophoresis trapping force applied in combination with the flow rate and is inevitably happening using a force strong enough to ensure a high trapping efficiency. It was observed that this phenomenon was more frequent in the beginning of the trapping area^36^. Despite of this it was never challenging to identify and count the trapped cells as the shallow wells will not allow cells to be trapped directly above each other.

**Figure 6:**
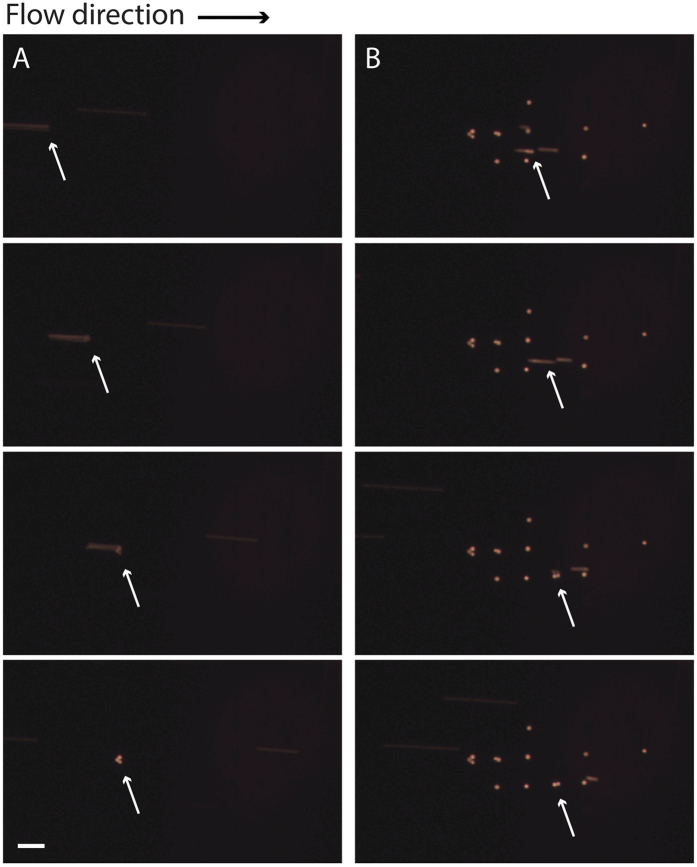
Time lapse of trapping of separated cancer cell clusters (orange) of (**A**) three or (**B**) two cells observed and indicated by the white arrows. Scale bar indicates 100 μm.

It was also seen that some rarely occurring clusters of cancer cells were efficiently trapped, even though the array and well designs were optimized for single cells (Fig. 6). It is expected that any occurring CTC cluster will be deflected to the target outlet in the acoustophoresis chip and enter the trapping zone easier than single cells as these clusters are larger and the acoustophoresis separation mechanism is predominantly size dependent. The more cells in the cluster, the easier it will be deflected by the acoustic force and enter the trapping zone. Importantly, this show great promise for the usefulness of the presented microfluidic system since CTC clusters, or microemboli, have been suggested to have a significantly greater metastatic potential than individual cells^40^. Sarioglu *et al*.^41^. previously proposed a microfluidic device for capturing these clusters. Their device was, however, not able to trap single-cell CTCs as the system reported herein is.

### Separation, concentration, and trapping of live cancer cells from whole blood-concentrated PBMC

**Figure 7:**
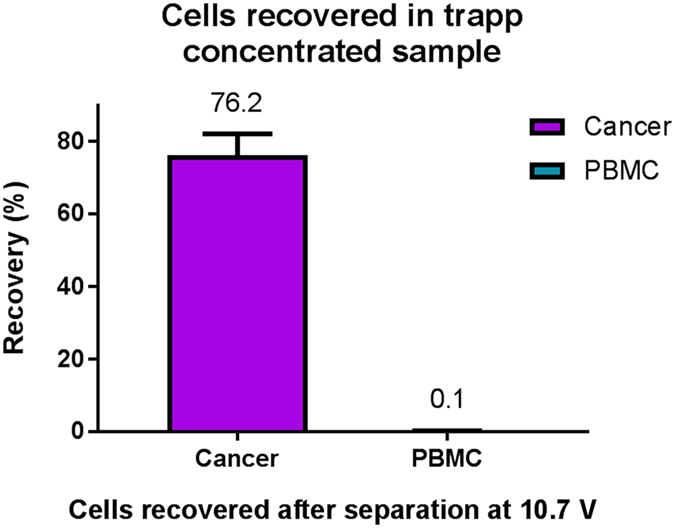
Percentage of cells recovered in the trap after acoustic separation of cancer cells from PBMC at a physiological concentration, as compared to the concentration of the input sample. The error bars show the standard deviation.

After characterizing the device with a dilute concentration of cells a sample with a physiological concentration of the viable PBMCs together with spiked viable cancer cells was run through the device and the most optimal voltage setting of the acoustic separation transducer was chosen. The sample contained ~2 000 000 PBMCs/mL and ~7500 cancer cells/mL. The result was obtained in the same way as the previously described experiments with the dilute samples. The result is shown in Fig. 7 where it can be seen that at these settings it was possible to recover 76.2 ± 5.9% of the cancer cells while at the same time trapping 0.12 ± 0.04% PBMCs. The recovery of PBMCs corresponds to the trapping of between 2917–1550 cells per mL processed sample. Although run at the same voltage setting as for the dilute sample the recovery of both cancer cells and PBMCs are slightly higher for this more highly concentrated sample. This can be explained by a small instability in the system that might be seen between runs it may also be a result of hydrodynamic coupling between the cells as they are translated by the acoustic force towards the center of the acoustic separation zone, which in turn results in more cells entering the dielectrophoretic trapping zone. In order to process a whole blood sample of 5 mL without filling up the trap completely with contaminating PBMCs the acoustic separation voltage will either have to be lowered, which will also result in a slightly lower recovery of cancer cells, or the number of wells will have to be increased further. A two-fold increase of wells would be able to accommodate the current PBMC contamination observed.

The results presented herein hold promise for further development towards the isolation of CTCs in clinical samples. The acoustic pre-alignment enables an efficient separation, while the acoustic concentration step enables the integration with the dielectrophoretic trapping chip. The integration of the trapping function together with the separation function effectively eliminates the post-separation processing needed before the sample analysis, which reduces the risk of sample loss and shortens the analysis time needed. At the current sample input flow rate the device can process a sample of 4.8 mL in an hour, a throughput that is well within a diagnostic and physiologically relevant range.

In order to increase the throughput further the pre-alignment zone can be elongated in expense of the separation channel. In the pre-alignment zone the initially randomly positioned cells will all have to be acoustically transferred into the two acoustic pressure nodes, while they, in the separation zone, only have to be partly sideways deflected, and not fully focused into the channel center, in order to enter the concentration zone.

In order to increase the separation efficiency further the acoustic impedance of the DEP buffer can be manipulated. At its current formulation, the acoustic impedance of the buffer is higher (data not shown) than for PBS that has previously been commonly used as a carrier medium for acoustic cell separation applications since large amount of sucrose (236 mM) was added to the DEP buffer to adjust the osmotic pressure. As the relative mobility of the cells in the acoustic field is dependent on the cell’s properties compared to the properties of the carrier medium, a medium with higher acoustic impedance might induce a change in the relative mobility between the different cell populations to be separated. It has previously been seen that *e.g.* neutrophils, the most abundant granulocyte population, has a higher acoustic impedance than the breast cancer cell line MCF7^42^, which have previously been observed to show a similar acoustic mobility to the DU145 prostate cancer cell line used in this work^38^. Effectively this means that in a medium with higher acoustic impedance the neutrophils will display a higher relative acoustic mobility compared to the cancer cells and the separation will thus not be as efficient as in a medium with lower acoustic impedance. Even though the red blood cells where depleted from the white blood cells through gradient density centrifugation, where most granulocytes will also be depleted as they inherently have a higher density than the mononuclear cell fraction of the white blood cells, some of these may still be present in the processed sample. These cells will then likely be among the white blood cells with the highest acoustic mobility that will be the first to translocate together with the separated target cells into the trapping zone contaminating the target cell fraction.

The current device relies on pre-staining of the cells. Ongoing work targets this issue and in-trap based staining is an evident way forward. Previous work on cell manipulation in the DEP-trapping array has demonstrated several in-trap performed bioanalyses such as immunostaining, viability assay, and fluorescent *in situ* hybridization (FISH) at the single-cell level just by applying specific reagents for each assay^36^.

## Conclusions

In this paper an integrated device combining acoustophoresis and dielectrophoresis for label-free separation, concentration, and trapping of cancer cells is presented. Cells of physiological concentrations can be effectively separated and trapped for direct enumeration, identification, and analysis of the target cells, effectively reducing the analysis time and eliminates the risk of sample loss during sample preparation for analysis post-separation. Future work will be focused towards developing more advanced on-chip labelling and analysis methods for the trapped single cell array.

## Materials and Methods

### Device design and chip fabrication

#### Acoustic chip

**Figure 8:**
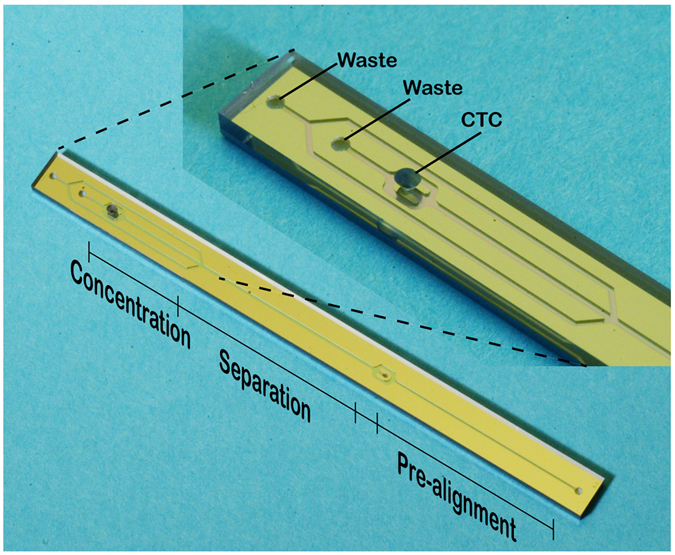
The acoustophoresis pre-alignment, separation and concentration chip.

The acoustic chip design was optimized from an earlier chip design^38^, and fabricated by Micronit Microtechnologies B.V. (Enschede, The Netherlands). The chip has two inlets, a sample inlet and a sheath fluid inlet, and three outlets, a separation waste outlet, a concentration waste outlet and a sample outlet subsequently leading to the dielectrophoresis trapping chip. The sample inlet leads to the pre-alignment zone, with a width of 300 μm and height of 150 μm, and a length of 2.3 cm. The sheath fluid inlet follows and enters the separation zone with dimensions 375 μm width and 150 μm height, and a length of 2.3 cm. After leading off the separation waste to the separation waste outlet the target sample enters the concentration zone also of dimensions 375 μm and 150 μm for width and height, respectively, and a length of 1 cm, Fig. 8. The pre-alignment zone was actuated using 5 MHz and 34 V and the separation and concentration zones were actuated using 2.08 MHz and varying voltages. The actuation was performed using piezoceramic transducers (PZ26, Ferroperm piezoceramics, Kvistgaard, Denmark). Furthermore, the chip temperature was controlled using a Peltier element (Farnell, London, UK) and a Pt1000 temperature sensor (Farnell, London, UK).

#### Dielectrophoresis chip

The single-cell trapping EMA chip was modified from the previous chip to improve cell-trapping capacity, and fabricated using conventional photolithography and etching processes^35^. The shape of the electrodes was patterned on an ITO-coated glass substrate (GEOMATEC co., Japan) by using a positive-type photoresist (S1813; Shipley far Ltd., USA) and ITO was etched with an etchant (ITO-02; KANTO KAGAKU co., Japan) for 5 min at 40 °C. After removing the photoresist layer remaining on the substrate, the microwell array was fabricated with a negative-type photoresist (SU-8 3005; MicroChem Co., USA) on the ITO electrodes. To achieve single-cell resolution during cell trapping, the diameter of the microwells (24 μm) was comparable to that of the target cells and the thickness of the microwell was 4 μm to allow efficient cell trapping by reducing the cell-to-electrode distance^43^. To deliver the cells to the microwell array, a microfluidic channel was fabricated by PDMS (Silopt 184; Dow Corning Toray, Co. Ltd.) through a standard replica molding process^35^. A mold master was fabricated with a negative-type photoresist (SU-8 3035, MicroChem Co., USA) with a thickness of 50 μm. A PDMS prepolymer was mixed with a curing reagent (at a 10:1 mass ratio), and poured into the mold master and cured at 75 °C for 2 h. After making holes for access ports, the PDMS channel and the microwell array substrate were exposed to O_2_ plasma using reactive-ion etching (RIE-10NR; Samco Co., Japan) and bonded together. For the DEP cell trapping, an electrical potential of 5 V at 5 MHz was applied to the electrodes, where a strong DEP force was achieved by applying a megahertz-range electric field.

#### Integration of devices

The integrated device was bonded together through plasma treatment of both the acoustic chip glass and the PDMS surface of the dielectrophoresis chip. The outlet hole of the acoustic chip was aligned with the inlet hole of the dielectrophoresis chip and permanently bonded together. The other inlets and outlets of the acoustic chip were accessed through the silicon on the back side of the chip.

#### Instrument setup

The actuation of both the acoustic and the dielectrophoresis chip, was performed by two dual-channel function generator (AFG 3022B; Tektronic UK Ltd., Bracknell, UK). Signals to the piezoelectric transducers were amplified using an in-house build power amplifier, based on an LT1012 power amplifier (Linear Technology Corp., Milpitas, CA, USA) and one commercial amplifier (AG Series Amplifier, T&C Power Conversion Inc, Rochester, NY, USA). The signal to the dielectrophoresis chip was not amplified. The applied voltages to the piezoelectric transducers were monitored using an oscilloscope (TDS 2120, Tektronix) and the temperature was controlled using a Peltier-controller (TC2812; Cooltronic GmcH, Beinwil am See, Switzerland). Flow rates were set using glass syringes (Hamilton Bondauz AG, Switzerland) mounted on syringe pumps (Nemesys, Cetoni GmbH, Korbussen, Germany) and the flow rates of all inlets and outlets were controlled. The sample and sheath fluid inlet flow rates were set to 80 μL/min, the separation waste outlet flow rate was set to 150 μL/min, the concentration waste outlet was set to 6 μL/min and the sample outlet leading to the dielectrophoresis trapping zone was set to 4 μL/min. The temperature of the acoustic chip was set to 40 °C throughout the experiment.

#### Microparticles

The optimal acoustic conditions were determined using 4 and 6 μm (diameter) FITC-labeled melamine microparticles (Sigma-Aldrich, Buchs, Switzerland).

#### Cell culture and blood samples

The prostate cancer cell line DU145 and was used for the experiments. The cell line was acquired from ATCC and cultured according to their guidelines. Blood was collected from healthy donors, with informed consent, at Skåne University Hospital in Lund, Sweden.

#### Cell preparations

Peripheral blood mononuclear cells (PBMCs) where isolated using density gradient centrifugation. Briefly, 9 mL of PBS was added to 6 mL of blood. The diluted blood was then gently layered over a density gradient medium (Lymphoprep, StemCell Technologies, Vancouver, Canada) and centrifuged for 30 minutes at 400 g without breaks and low acceleration. The mononuclear cell layer was then recovered and transferred to four 50 mL tubes filled up with PBS. These were then subsequently washed by centrifuging at 200 g for 10 minutes. After this the PBMCs were stained with either 1 μM CellTrace Oregon Green or 1 μM CellTracker Orange CMTMR Dye (both ThermoFisher Scientific, Waltham, MA, USA) according to the manufacturer’s instructions.

Cultured cancer cells were harvested with trypsin/EDTA and stained with either 0.5 μM CellTrace Oregon Green or 0.5 μM CellTracker Orange CMTMR Dye according to the manufacturer’s instructions.

A known number of cells were subsequently transferred to a DEP buffer to allow the right conditions for the dielectrophoresis trapping. The DEP buffer consisted of 10 mM Hepes, 0.01 mM CaCl_2_, 59 mM D-glucose, 236 mM sucrose, and 2% BSA, solved in MilliQ water, and was prepared freshly for each experiment.

#### Trapping efficiency experiment

The trapping efficiency experiments were preformed without the acoustic pre-sorting. Cell samples were infused into the trap at different flow rates, 16 μL/min, 8 μL/min, 4 μL/min, and 2 μL/min during a specified time when the cells that did not trap where counted in flow. The flow was then stopped and the cells trapped were counted. The number of cells that did not trap and those that were captured were then compared to obtain the trapping efficiency.

#### Separation experiments

Three reference cell concentrations for the PBMCs and the cancer cells, respectively, where obtained through counting the cell concentrations of the original cell solution using a FACS (FACS Canto II, BD Bioscience). A sample of known volume was then taken from the reference sample and transferred to the DEP buffer. A mean of the three samples was then calculated and compared to the number of trapped cells in each experiment to obtain the cell recovery.

## Additional Information

**How to cite this article:** Antfolk, M. *et al*. Label-free single-cell separation and imaging of cancer cells using an integrated microfluidic system. *Sci. Rep.* **7**, 46507; doi: 10.1038/srep46507 (2017).

**Publisher's note:** Springer Nature remains neutral with regard to jurisdictional claims in published maps and institutional affiliations.

## Supplementary Material

Supplementary Information

Supplementary Video

## Figures and Tables

**Table 1:** Summary of analytical performance of microfluidic devices enabling on-chip CTC analysis.

Method	Input sample	Flow rate	Recovery	Contamination/purity	Reference
Magnetic trapping targeting EpCAM	50–1000 cells /mL Colo-205 or SKBR3 cancer cells spiked into whole blood	167 μL/min	Up to 90% and 86%, respectively.	Not mentioned	28
Magnetic trapping targeting EpCAM	2–80 cells/mL M6C cancer cells spiked into RBC-lysed blood	20 μL/min	87%	Contamination < 0.4% WBCs	29
Magnetic trapping targeting EpCAM	50–200 cells/mL VCaP cancer cells in whole blood	33 μL/min	> 80%	Contamination < 0.035% measured from trapping U937 cancer cells in buffer solution	30
Inertia and microvortices	500 cell/mL MCF7 spiked into 5% blood	5 mL/min	20%	Purity 40%	31
Immunoaffinity targeting EpCAM	50–50000 cells/mL of NCI-H1650 cancer cells in whole blood	17–33 μL/min	>60%	Purity 50%	24
Immunoaffinity targeting EpCAM	1000 cells/mL of PCR cancer cells in whole blood	25–42 μL/min	92%,	Purity 14.0 ± 0.1%	32
Filter (pillar-type) capturing CTC clusters	MDA-MB-231 clusters in whole blood	42 μL/min	40–100% for 2− > 4 cancer cells in the cluster	Not mentioned	33
