# Supplementary material for: Label-free single-cell separation and imaging of cancer cells using an integrated microfluidic system
Source: Sci Rep. 2017 Apr 20;7:46507. doi: 10.1038/srep46507 (PMC5397835; doi:10.1038/srep46507)
Supplement: Supplementary Information [file srep46507-s1.pdf]

Supplementary Information

Label-free single-cell separation and imaging of cancer cells using an integrated microfluidic system

Maria Antfolk*, Soo Hyeon Kim*, Saori Koizumi, Teruo Fujii, Thomas Laurell

*Authors contributed equally

S1 The acoustic radiation force F_{rad}

$$F_z = -\partial_z U^{rad} = 4\pi\phi(\tilde{\kappa},\tilde{\rho}) k a^3 E_{ac} \sin(2kz) \qquad \text{Eq. 1}$$

$$E_{ac} = \frac{p_a^2}{4\rho_0 c_0^2} \qquad \phi(\tilde{\kappa},\tilde{\rho}) = \frac{1}{3}\left[\frac{5\tilde{\rho}-2}{2\tilde{\rho}+1} - \tilde{\kappa}\right] \qquad \tilde{\rho} = \frac{\rho_p}{\rho_0} \qquad \tilde{\kappa} = \frac{\kappa_p}{\kappa_0}$$

where F_z denotes the lateral acoustophoresis force, k is the wave number ($2\pi/\lambda$), E_{ac} is the acoustic energy density, a - particle radius, κ_0, ρ_0, κ_p and ρ_p are the compressibility and density of the fluid and particle, respectively and $\phi(\tilde{\kappa},\tilde{\rho})$ is the acoustic contrast factor, z is the position of the particle along the wave propagation axis, p_a is the pressure amplitude, c_0 is the speed of sound in the medium.

S2 Merged image of the EMA trapping area

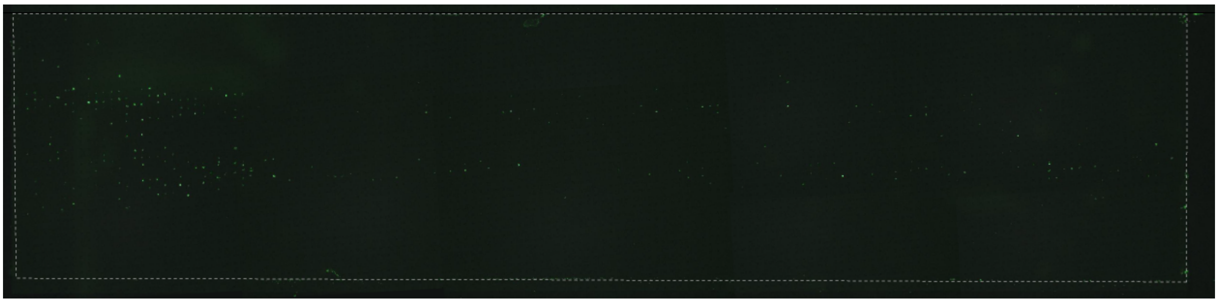

Figure S2. Merged image of the entire trapping area where cancer cells (green) can be seen trapped in some of the wells. The sample flow direction is from left to right in the image whereas more cancer cells are seen trapped in the first part of the trap.

S3 Video showing trapping of DU145 after separation.

The supplementary video shows trapping of DU145 prostate cancer cells after separation from peripheral blood mononuclear cells. The flow rate in the trap is 4 μL/min.
